# Correction to “hnRNPA2/B1 activates cyclooxygenase‐2 and promotes tumor growth in human lung cancers”

**DOI:** 10.1002/1878-0261.70016

**Published:** 2025-03-10

Xuan Y, Wang J, Ban L, Lu J‐J, Yi C, Li Z, Yu W, Li M, Xu T, Yang W, Tang Z, Tang R, Xiao X, Meng S, Chen Y, Liu Q, Huang W, Guo W, Cui X, Deng W. hnRNPA2/B1 activates cyclooxygenase‐2 and promotes tumor growth in human lung cancers. *Molecular Oncology* 2016;10(4):610–24. doi: 10.1016/j.molonc.2015.11.010.

In the article by Xuan *et al*., an error occurred during figure assembly resulted in Fig. 4A being published with a duplicated scratch assay, showing the same image for 0 h Ctrl and shRNA. In addition, scale bars were missing on the tumor samples in Fig. 5A and the microscopy images in Fig. 7B.

The authors have corrected these by providing the original raw data for all experimental replicates, and the revised figures are included here. All authors agree to this corrigendum and confirm that changes do not affect the conclusions of the article.

The corrected figures are reproduced below.

**Figure 4A**

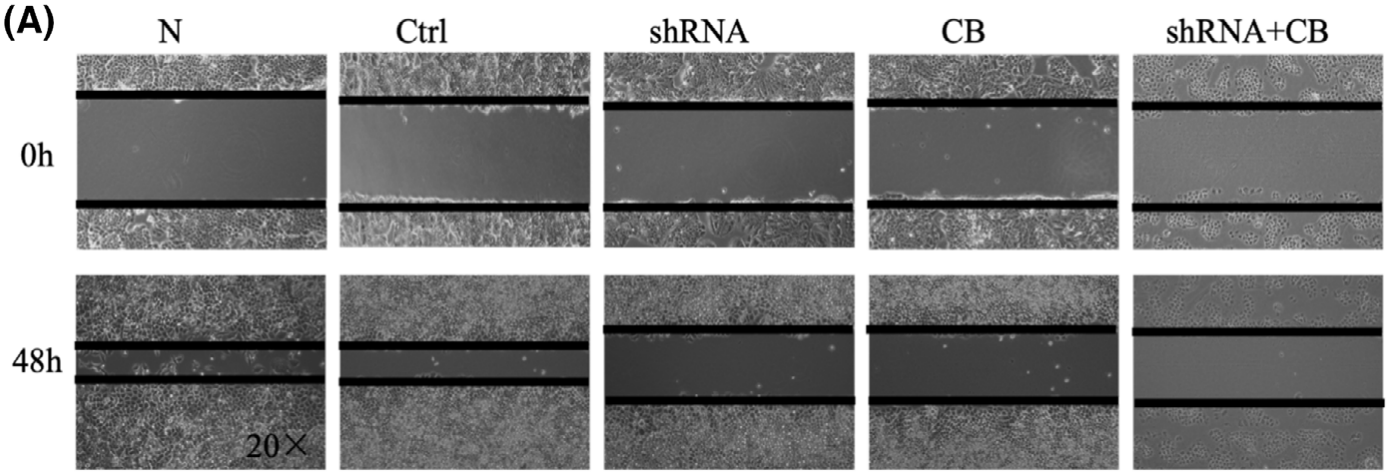

**Figure 5A**

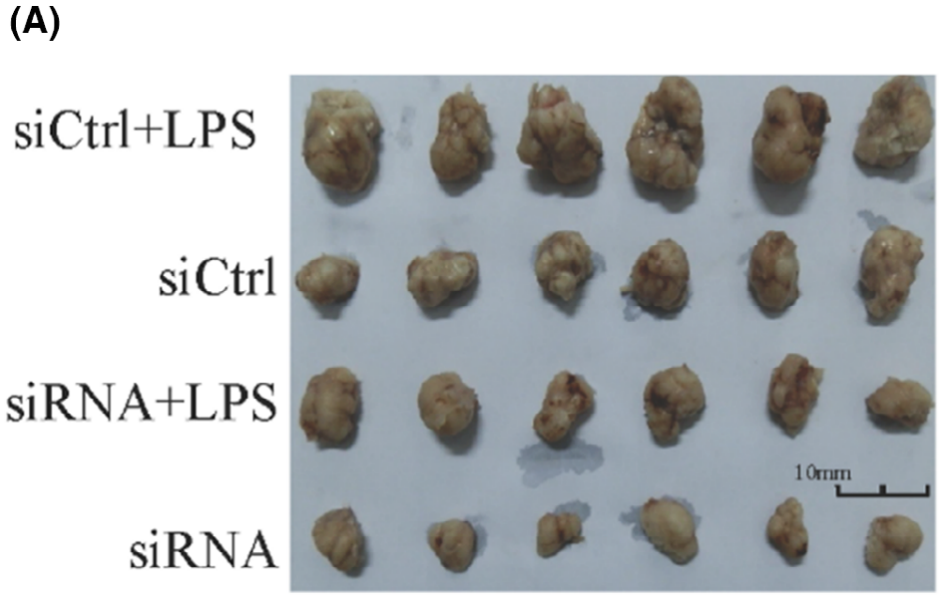

**Figure 7B**

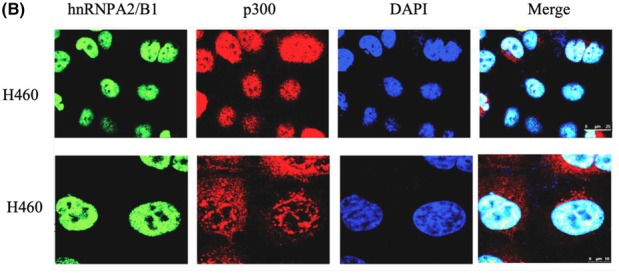

The authors apologize for any inconvenience caused.

